# Dense Convolutional Neural Network-Based Deep Learning Pipeline for Pre-Identification of Circular Leaf Spot Disease of *Diospyros kaki* Leaves Using Optical Coherence Tomography

**DOI:** 10.3390/s24165398

**Published:** 2024-08-21

**Authors:** Deshan Kalupahana, Nipun Shantha Kahatapitiya, Bhagya Nathali Silva, Jeehyun Kim, Mansik Jeon, Udaya Wijenayake, Ruchire Eranga Wijesinghe

**Affiliations:** 1 Department of Computer Engineering, Faculty of Engineering, University of Sri Jayewardenepura, Nugegoda 10250, Sri Lanka; deshankalupahana@sjp.ac.lk (D.K.); egt18538@sjp.ac.lk (N.S.K.); 2 Department of Information Technology, Faculty of Computing, Sri Lanka Institute of Information Technology, Malabe 10115, Sri Lanka; nathali.s@sliit.lk; 3 Center for Excellence in Informatics, Electronics & Transmission (CIET), Sri Lanka Institute of Information Technology, Malabe 10115, Sri Lanka; 4 School of Electronic and Electrical Engineering, College of IT Engineering, Kyungpook National University, 80 Daehak-ro, Buk-gu, Daegu 41566, Republic of Korea; jeehk@knu.ac.kr (J.K.); msjeon@knu.ac.kr (M.J.); 5 Department of Electrical and Electronic Engineering, Faculty of Engineering, Sri Lanka Institute of Information Technology, Malabe 10115, Sri Lanka

**Keywords:** circular leaf spot (CLS) disease, classification, deep learning (DL), disease identification, optical coherence tomography (OCT), transfer learning

## Abstract

Circular leaf spot (CLS) disease poses a significant threat to persimmon cultivation, leading to substantial harvest reductions. Existing visual and destructive inspection methods suffer from subjectivity, limited accuracy, and considerable time consumption. This study presents an automated pre-identification method of the disease through a deep learning (DL) based pipeline integrated with optical coherence tomography (OCT), thereby addressing the highlighted issues with the existing methods. The investigation yielded promising outcomes by employing transfer learning with pre-trained DL models, specifically DenseNet-121 and VGG-16. The DenseNet-121 model excels in differentiating among three stages of CLS disease (healthy (H), apparently healthy (or healthy-infected (HI)), and infected (I)). The model achieved precision values of 0.7823 for class-H, 0.9005 for class-HI, and 0.7027 for class-I, supported by recall values of 0.8953 for class-HI and 0.8387 for class-I. Moreover, the performance of CLS detection was enhanced by a supplemental quality inspection model utilizing VGG-16, which attained an accuracy of 98.99% in discriminating between low-detail and high-detail images. Moreover, this study employed a combination of LAMP and A-scan for the dataset labeling process, significantly enhancing the accuracy of the models. Overall, this study underscores the potential of DL techniques integrated with OCT to enhance disease identification processes in agricultural settings, particularly in persimmon cultivation, by offering efficient and objective pre-identification of CLS and enabling early intervention and management strategies.

## 1. Introduction

*Diospyros kaki*, more generally referred to as the Japanese persimmon, is a fruit that is native to a number of countries and regions, including Spain, China, South Korea, and Japan. This plant possesses considerable importance due to its status as one of the earliest cultivated plants on a worldwide scale, being highly regarded for its nutritional content and therapeutic properties. Among persimmon-related diseases, *Mycosphaerella nawae*, which causes circular leaf spot (CLS) disease, stands out as one of the most devastating diseases [1]. CLS is characterized by the discoloration and defoliation of persimmon leaves, resulting in the premature abscission of the fruit [2]. The widespread prevalence of CLS poses significant economic challenges in regions where persimmons are cultivated. Detecting and controlling CLS disease in its nascent stages proves daunting due to the complexities involved in its identification based on external characteristics.

The extended incubation period of CLS disease can last anywhere from 90 to 120 days. Thus, earlier detection of infection will help in limiting the harm that the disease can cause [3]. Farmers traditionally rely on manual inspection of leaf thickness as an initial method of identifying CLS disease before visible symptoms manifest. However, such identification methods necessitate significant experiential expertise and remain inherently subjective. Polymerase chain reaction (PCR) serves as the gold standard for pathogen identification, offering direct detection capabilities [4]. Nonetheless, its implementation in the field is hindered by the destructive nature of sample preparation and time-consuming procedures. Visual inspection techniques, including scanning electron microscopy (SEM), transmission electron microscopy (TEM), light microscopy, and magnetic resonance imaging (MRI), have been utilized for plant disease identification by observing morphological changes [5]. However, microscopy techniques require destructive sampling, while the resolution of MRI has challenges in distinguishing diseased and healthy tissues [6,7].

To address challenges associated with both direct identification techniques, such as PCR and indirect visual inspection methods, optical coherence tomography (OCT) emerges as a promising alternative by offering several advantageous features, such as real-time operation and non-invasive inspection and visualization with a micrometer resolution, which are highly beneficial for agricultural inspections [8,9,10,11]. This non-invasive OCT technology offers high-resolution images at the micrometer scale in real-time. Numerous studies have been directed towards leveraging OCT for CLS identification, employing it as an indirect method to analyze leaf morphology for disease detection. However, accurate interpretation of OCT data often necessitates specialized knowledge and expertise in plant pathology, which may not be widely available.

In response, the application of deep learning (DL) techniques has gained traction for automated analysis of optical imaging across various domains [12]. This approach enables efficient analysis while circumventing the limitations associated with expertise in image interpretation, thereby enhancing objectivity in detection.

Building on this trend, this study aimed to enable early detection of CLS disease in persimmons while addressing the limitations of current CLS identification methods in agricultural settings. It involved creating a specific dataset and developing optimized DL models for efficient, advanced identification of CLS. The significance of this study can be summarized as follows:

- Developed a DL-based framework using optimized transfer learning models for the automated pre-identification of CLS disease in persimmons, addressing the time-consuming and subjective nature of traditional detection methods.
- Developed a highly curated dataset for CLS classification using manual analysis of OCT amplitude scans (A-scan) and loop-mediated isothermal amplification (LAMP), focusing on pre-identification of CLS.
- Presented the inaugural application of OCT combined with DL for disease detection in plant leaves within agricultural contexts.
- Utilized a novel combination of LAMP with A-scan for detecting CLS disease in persimmon.

## 2. Related Works

**Figure 1:**
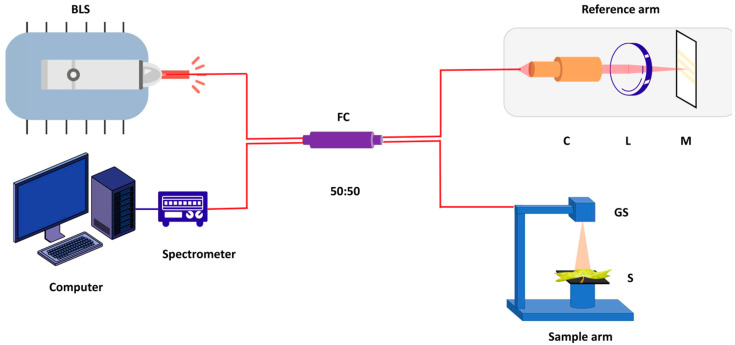
Schematic of the spectral domain optical coherence tomography system (SD-OCT), highlighting the reference arm, sample arm, spectrometer, computer and key components such as the broadband light source (BLS), fiber coupler (FC), collimator (C), lenses (L), mirror (M), sample (S) and galvanometer scanner (GS).

OCT gained prominence following the publication of a seminal paper by Huang et al. in 1991, which focused on the non-invasive cross-sectional imaging of biological systems [13]. This work demonstrated the application of OCT for imaging the retina within the human eye. Subsequent to this, advancements and widespread applications of OCT in acquiring images of the human body have been reported [14,15,16,17,18,19]. In the OCT scanning system illustrated in Figure 1, the optical light beam from the broadband light source splits into the reference and sample arms using an optical fiber coupler. A galvanometer-based optical scanner performs the sample scanning. The reflected signals from the sample and reference arm get interfered with by the beam coupler and forwarded to a spectrometer. The attached computer system will create the image of the sample from the data acquired through the spectrometer. This mechanism yields high-resolution cross-sectional images of biological tissues [20].

The OCT images can be assessed quantitatively and qualitatively using amplitude scans (A-scans), two-dimensional OCT (2D-OCT) images, and volumetrics. A-scans provide insights into the reflectivity or amplitude of light as a function of depth within the imaged tissue. B-scans generate a 2D cross-sectional image of the imaged tissue, while volumetric scanning produces a three-dimensional (3D) representation of the scanned sample.

In addition to being non-invasive and non-destructive, OCT is able to produce high-resolution images at the micrometer scale, making it an ideal diagnostic tool for a wide range of plant-related scientific endeavors. Wijesinghe et al. conducted a study on plant disease identification, focusing on analyzing morphological changes in infected tissues throughout the incubation period [3]. The research team developed a wearable backpack-type OCT system equipped with a customized spectral domain OCT (SD-OCT) for on-site inspections for grey leaf spot disease in *Capsicum annuum* and apple blotch in *Marssonina coronaria*, while a swept source OCT (SS-OCT) system was employed for identifying CLS disease in persimmon [11,21,22]. Also, a method was proposed by the same group for the early detection of CLS disease utilizing OCT imaging [3]. The primary aim of their study was to introduce a protocol for detecting CLS disease by assessing the reduction in thickness of the palisade parenchyma cell layer in leaf cross-sections, thereby curtailing disease spread. Furthermore, a histological examination was carried out to better understand the connection that exists between OCT images and the intended biological region of interest.

DL is a subset of machine learning (ML) that employs artificial neural networks (ANN) with multiple layers of interconnected nodes, allowing the system to learn intricate patterns and representations directly from raw data. It has revolutionized various fields, including computer vision, natural language processing, and speech recognition, by enabling the development of highly accurate and efficient models for complex tasks [23,24,25]. DL algorithms excel at automatically extracting features from large datasets, making them particularly well-suited for tasks such as image classification, object detection, and language translation. With advancements in hardware and algorithms, DL has become increasingly prevalent in industries ranging from healthcare to autonomous vehicles and robotics, driving innovations and transformative solutions across diverse domains [26,27].

The utilization of DL methods has witnessed a growing trend in the field of medical imaging, specifically for the automated analysis of medical images [28]. OCT has been shown to have a wide range of applications in the diagnosis of ocular diseases. There have been numerous studies that have been conducted to investigate these diseases by employing OCT in conjunction with DL approaches [29,30,31,32,33,34,35]. For instance, Karry et al. utilized a publicly available OCT dataset in 2017 to classify and recognize age-related macular degeneration (AMD) and diabetic macular edema (DME) from OCT images, achieving satisfactory results by employing GoogleLeNet a Convolutional Neural Network (CNN) developed by Google for the ImageNet competition [36]. In 2019, Wang et al. applied DL techniques to diagnose AMD and DME using two publicly available datasets. They trained a novel CNN model named CliqueNet and attained a high classification accuracy exceeding 98% for both datasets, comparing favorably with transfer learning methods utilizing architectures such as VGG16, VGG19, and ResNet50 [37]. Furthermore, Kugelman et al. employed a DL model combining CNN and a Recurrent Neural Network (RNN) for automatic choroidal segmentation in OCT images [38]. Despite the relatively limited use of OCT in agriculture, there have been notable attempts to apply DL in agricultural contexts for OCT analysis. Joshi et al. explored a novel approach for classifying rice seeds using OCT in conjunction with a Deep Neural Network (DNN), eliminating the need for labels and invasive procedures [39]. Likewise, Manhando et al. introduced an innovative framework aimed at early identification of mold-contaminated peanut kernels in the post-harvest phase, utilizing OCT images [40].

## 3. Materials and Methodology

**Figure 2:**
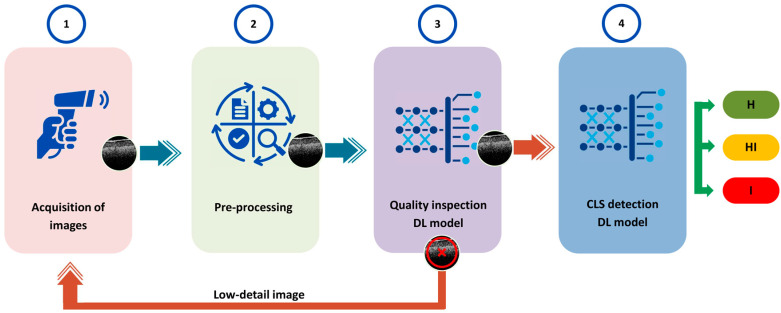
Deep learning (DL) based pipeline for pre-identification of circular leaf spot (CLS) disease of persimmon. Class H, HI and I denote healthy, healthy-infected and infected, respectively.

The proposed pipeline, based on DL, aims to automatically pre-identify CLS disease in persimmon leaves, as illustrated in Figure 2. Initially, OCT B-scan images of leaf specimens are captured using a scanning device. Preprocessing is performed on these images in order to prepare them for subsequent analysis. Subsequently, the preprocessed images are fed into the first DL model, which determines the suitability of the image samples for CLS disease prediction. This model works as a binary classifier that outputs true for suitable images and false for images that lack quality. When images lack sufficient details, processing will be paused to facilitate either rescanning or moving to the next sample. Conversely, images with adequate detail progress to the next stage, where the second DL model is employed for classification. This model categorizes samples as infected (class-I), apparently healthy or apparently infected (class-HI-healthy infected), or non-infected (class-H-healthy).

### 3.1. Experimental Setup and Data Acquisition

#### 3.1.1. Collection of Plant Materials

All of the persimmon leaf specimens were collected from a persimmon orchard located in Sangju in Gyeongbuk province, Korea. The entire persimmon orchard with an annual yield is treated with fungicides and insecticide during cultivation. A total number of ten trees were selected and the sample collection was conducted in a random process. The entire experiment was carried out once a week from early May to late September. During the experimental period, 5 leaf specimens were selected from each tree on experimental day, while examining a total of 1000 leaf specimens at 20 attempts.

#### 3.1.2. System Configuration

The OCT image acquisition was conducted using a laboratory customized backpack type wearable OCT system. The OCT engine consists of a broadband light source consisting of a superluminescent diode (EXS210068-01, Exalos, Schlieren, Switzerland) with a center wavelength of 850 nm and a bandwidth of 55 nm. A line scan camera was used as the detector (spL2048-140 km, Basler, Ahrensburg, Germany). All of the components were precisely calibrated to enhance the axial resolution. The scanning depth of the system was measured as 3 mm, while the axial and lateral resolutions were measured as 8 μm and 12 μm. Detailed technical specifications can be found in [10,21].

The OCT signal fluctuation along the axial direction as well as the quantitative evaluation procedure fundamentally depends on the refractive index of the structure, since the refractive index of each deep layer differs from one to another. Although each layer in each depth level has a unique refractive index, a refractive index of 1.42, which is the fundamental refractive index of plant cells, was used herein. Therefore, once the particular refractive index of each layer is applied to the acquired 2D-OCT images, the corresponding most accurate axial direction depth profile can be obtained. Since the employed refractive index 1.42 provides sufficient visualization of the inner morphology, this value was used for the entire depth range.

#### 3.1.3. LAMP Technique

To identify a clear physiological stage, all of the collected leaf specimens underwent LAMP examination followed by the OCT inspection. During LAMP examination, the collected leaf surface was sterilized. Next, these leaves were extracted using a mesh sample bag (Agdia, Elkhart, IN, USA) and tissue homogenizer (ACC00900, Agdia) with extraction buffer. Then, 1 µL of extraction buffer was added to a LAMP mixture, where the mixture content can be found in [22]. Afterwards, the mixture was incubated at the optimized temperature using a digital water bath (JSWB-22T, JSR, Gongju-City, Korea) for 5 min. Next, 1 µL of Bst polymerase large fragment (8 unit/µL, New England Biolabs, Ipswich, MA, USA) was added, and the samples were incubated at the optimized temperature for 1 h. A description of the overall LAMP process can be found in [22].

#### 3.1.4. Dataset

The dataset comprised 1500 2D-OCT images obtained from the OCT system, with the scanning area predominantly centered on regions near the midrib of the leaf samples. Each image possessed a resolution of 500 × 200 pixels.

### 3.2. Pre-Processing

**Figure 3:**
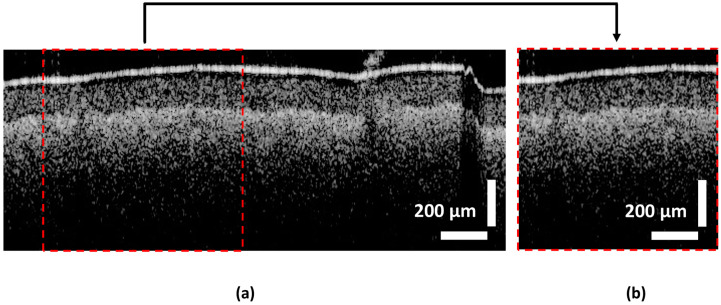
OCT images acquired from scanning the leaf samples of persimmon. (**a**) Original OCT image acquired with 500 × 200 pixels of size. (**b**) The image cropped from the original OCT image. Respective cropped position is shown in the original image.

The original dataset comprises 1500 OCT B-scan images of leaf samples. DL models necessitate a larger number of training samples to effectively learn and generalize patterns from data [41]. Additionally, DL models typically operate optimally with square matrices due to computational considerations and network architecture constraints. To augment the dataset, a single image measuring 500 × 200 pixels was cropped at three distinct horizontal pixel locations, specifically at the 50th, 100th, and 150th positions, resulting in cropped images of dimensions 200 × 200 pixels each. The choice of cropping positions ensured that the cropping window remained below the 400th horizontal pixel to mitigate the presence of noise, commonly observed in all images beyond this threshold, which could adversely impact the learning process of the models. This augmentation process yielded a total of 4500 images. Figure 3 depicts the original OCT image obtained from a persimmon, as well as a cropped image derived from the original image.

**Figure 4:**
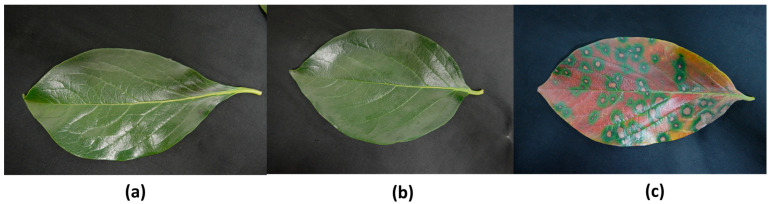
Photographs of the persimmon leaves from the infected and healthy trees. (**a**) Leaf from a healthy tree. (**b**) Healthy-looking leaf from an infected tree. (**c**) Infected leaf.

Two distinct datasets were created to facilitate the demands of the two separate DL models of the pipeline. Due to the presence of low-detail images within the dataset, a new balanced dataset was constructed using all low-detail images and randomly selected high-detail images. This refined dataset was employed to train the initial DL model responsible for identifying high-quality images to be fed into the second DL model in the fourth stage of CLS identification, as depicted in Figure 2. For training, the second dataset was utilized, which contained 3747 images after removing low-detail images and images with label ambiguities. Since horizontal flipping does not affect OCT images in this use case, all selected images were horizontally flipped to augment the dataset, resulting in a total of 7494 samples. This augmented dataset encompassed 1872 healthy (class-H) images, 5160 healthy-infected (class-HI) images, and 462 infected (class-I) images. Figure 4 shows example persimmon leaf specimens falling into each category.

Speckle noise, characterized by random patterns of bright and dark speckles overlaying images, is a common occurrence in OCT imaging. This noise originates primarily from the scattering of light in multiple directions and the resulting constructive and destructive interference during the scanning process [42]. To mitigate the effects of speckle noise, the Block-matching and 3D filtering (BM3D) algorithm was employed to effectively reduce its presence to an acceptable level.

### 3.3. Labeling of Datasets

**Figure 5:**
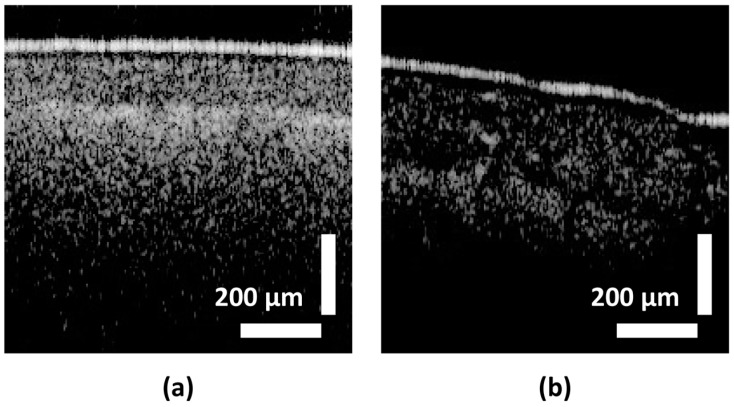
Sample OCT-B scan images utilized for training the quality inspection model. (**a**) High-detail image. (**b**) Low-detail image.

The initial dataset, comprising high-detail and low-detail images, was curated manually by meticulously examining all 4500 images. In the low-detail images, the tissue layers of the B-scan images of leaf samples were notably less discernible, rendering the identification of CLS disease more challenging. The dataset for the quality inspection model was generated based on the aforementioned dataset. All low-detail images were manually filtered out to ensure a balanced dataset, with an equal number of samples selected from the high-detail images. The resulting dataset comprised a total of 580 OCT B-scans (290 low-detail and 290 high-detail images). Figure 5 illustrates the disparities between a high-detail B-scan image and a low-detail B-scan image.

Several procedures were undertaken to label the dataset for the second model. LAMP represents a gold standard method for identifying plant pathogens. The original dataset was labeled based on LAMP tests that were carried out on leaf samples that were gathered from persimmon fields. These labeled leaf samples served as the ground truth for the labeling process. However, this can only detect whether the leaf sample is affected with the pathogen or not, dividing the samples into two classes. Pre-identification necessitates the detection of intermediary stages in order to allow for prompt treatment of plants prior to CLS spreading widely across the leaf.

**Figure 6:**
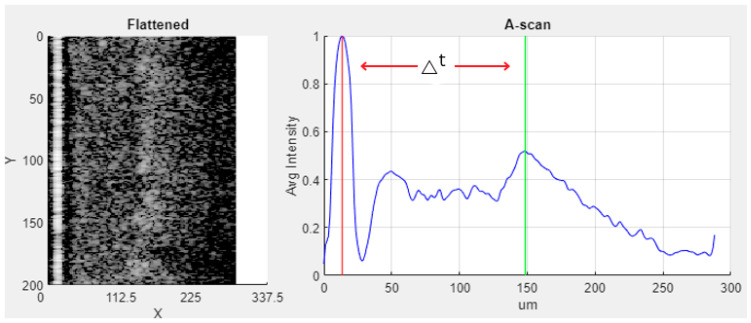
A-scan profile generated for the OCT B-scan image. The peaks denote the epidermal layer and parenchyma layer. The distance between the two peaks (∆t) was used as the factor to determine the class label according to Wijesinghe et al. [3].

The study undertaken by Wijesinghe et al. presents a methodology for differentiating between leaves that are in a healthy state, leaves that are looking healthy but infected, and leaves that are infected [3,43]. According to their analysis, infected leaves exhibit a thinner profile compared to healthy leaves. This differentiation is achieved by generating an A-scan profile from a corresponding B-scan image. Figure 6 illustrates the respective B-scan image alongside the generated A-scan image. The A-scan image depicts the mean intensity levels along the leaf’s surface as the scanner moves from the uppermost layer to the lowermost layer. The A-scan profile provides graphical representation of the different sub-layers present in the scanned leaf. The presence of CLS leads to a decrease in the separation between these sub-layers. This study suggests a strategy that utilizes the distance between two important layers, namely the epidermal layer and the spongy parenchyma layer, to simplify the process of labeling. In the A-profile, these layers exhibit the highest peaks, and the distance between these peaks serves as the basis for labeling class H, HI, and I samples. The labeling procedure is detailed in Table 1.

A MATLAB program was created to produce cropped images. Additionally, it was enhanced with a feature to generate A-scan profiles from these cropped images. Subsequently, it calculates the distance between the top two peaks in micrometers of the A-scan profiles. However, certain images presented challenges, such as unclear identification of peaks and the presence of multiple peaks unrelated to the calculation of interest. Consequently, all automatically labeled images underwent manual inspection using a dedicated MATLAB Graphical User Interface (GUI) program designed specifically for this purpose. This GUI comprises the label assigned by the automated labeling algorithm and respective LAMP of the sample. Manual labeling occurs through a comparison between these two values and the calculated peak difference. The label for a sample was manually assigned in cases of mismatch between the ground truth and the calculated label. If ambiguity persists between the ground truth and the label derived from the peak difference calculation, the respective images were excluded. The manual labeling procedure is outlined in Table 2.

### 3.4. Deep Learning Models

**Figure 7:**
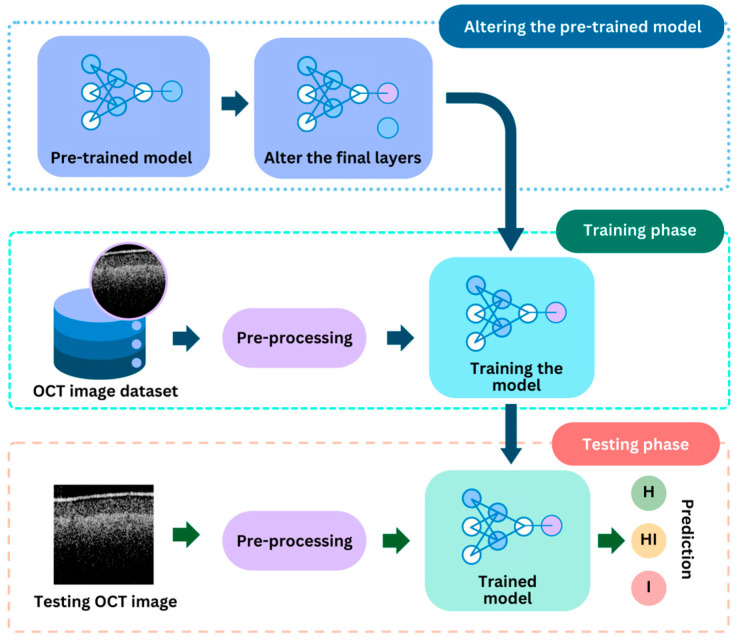
The process of training two deep learning (DL) models using transfer learning.

DL, which operates through ANN, has significantly revolutionized image identification and classification compared to alternative methods. Its capacity to automatically discern features from unstructured data by adjusting parameters and leveraging previously trained layers has enabled its widespread utilization in various analyses, particularly in morphological image studies [44]. Although the process of training DL models is time-consuming, the resulting trained models can be efficiently utilized in real-time to detect diseases. The pipeline’s process, which incorporates two DL models, is illustrated in Figure 7.

In this study, VGG-16 [45] was chosen as the optimal base model for the quality inspection model, which aims to classify high-detailed and low-detailed images. DenseNet-121 [46] was similarly selected as the most suitable base model for classifying samples into the categories of healthy (class-H), healthy-infected (class-HI), and infected (class-I). These models were preferred over a selection of other pre-trained models, including VGG-19, ResNet-50 [47], Inception3 [48], and Inception-ResNet-v2 [49].

DL models, such as VGG-16 and DenseNet-121, typically require a substantial number of training samples to reach an acceptable level of accuracy. However, the dataset collected for this study was insufficient to train all of the parameters of these models adequately. Consequently, transfer learning was employed to mitigate the impact of the limited dataset size. This approach has been widely utilized across various domains where large datasets are not readily available for training DL models [44,50]. The two pre-trained models employed in this study, namely VGG-16 and DenseNet-121, underwent pre-training using the ImageNet dataset, which encompasses 1.2 million color images spanning 1000 categories [51]. Subsequently, the final layer of each model was omitted, and a set of additional convolutional layers was introduced, followed by a set of fully connected layers for classification purposes. During the training process, the learning parameters of the original base model (VGG-16 or DenseNet-121) remained fixed, while the introduced parameters were permitted to be learned through the iterative training.

Both models were created using the Python programming language and the Google TensorFlow (v2.10.0) framework. These models share a similar code base, with variations made to accommodate different datasets, model types, and evaluation parameters. The models underwent training within a system equipped with specific hardware specifications, including a processor (Core i9-14900K, Intel, Santa Clara, CA, USA) having 24 physical cores and 32 threads, accompanied by 32 GB of memory. Additionally, the system was enhanced with a graphical processing unit (GPU) (GeForce RTX 4070, NVIDIA, Santa Clara, CA, USA) featuring 12 GB of memory, 5888 CUDA cores, and capable of delivering 568 Artificial Intelligence Trillions or Tera Operations per Second (AI TOPS).

### 3.5. Deep Learning Models for Quality Inspection

The quality inspection model was developed as a supplementary tool to enhance the detection accuracy of the primary objective of the study, which is the pre-identification of CLS. The dataset utilized for this model comprised 590 images, divided into training, validation, and testing subsets in a 3:1:1 ratio. Although the original image size was 200 × 200, it was scaled to 224 × 224, the default input dimension for most pre-trained models. The decision to use pre-trained models was driven by the limited number of samples.

The DL model is a customizable approach, allowing for easy adjustment of the backbone model, Fully Connected Network (FCN) layers, and other hyperparameters. In the best-performing model, all convolutional layers of the backbone model were frozen, and the FCN was connected by removing the initial FCN. The learning parameters of the FCN were randomly initialized for training purposes. This model performs binary classification to distinguish between high and low detailed images, using sigmoid activation in the final layer. Categorical cross-entropy loss was chosen as the loss function due to its efficacy with categorical data, and the optimizer used was stochastic gradient descent (SGD).

The model exhibited a tendency to overfit with a low number of iterations. Therefore, the number of layers in the FCN, dropout values in the layers, and L1 and L2 regularization were primarily tested to develop the best-performing model. The final optimal model included two layers for the FCN, with a hidden layer dropout value of 0.2 and an output layer configured with a kernel regularizer value of 0.04. This configuration resulted in the lowest bias and the best variance during training and testing. The quality inspection model was evaluated based on its overall accuracy and loss, given the balanced nature of the dataset.

### 3.6. Deep Learning Model for Identification of Circular Leaf Spot Disease

The CLS detection model is a crucial component of this study, inheriting similar development properties from the quality inspection model. Transfer learning was selected for this model due to its superior accuracy and generalization in classification compared to custom-built models. Nine pre-trained models were employed in this investigation: VGG-16, VGG-19, Inception-V3, Inception-ResNet-V3, ResNet-101, ResNet-50, EfficientNetB0, Xception, and DenseNet-121.

This model also utilizes input images of size 224 × 224, as square matrices offer benefits to CNNs by simplifying the application of filters in convolutions, efficiently sharing parameters across the image through the grid structure and standardizing the training data by establishing a uniform input format from images with different aspect ratios. The original backbone’s FCN was removed, and a new FCN was appended for CLS identification. Various foundational configurations of the FCN were explored in this study, with ReLU activation in the hidden layers and SoftMax activation in the output layer. Throughout the different phases of the study, various model variations were developed based on these foundational architectures by altering hyperparameters such as learning rate, batch size, and regularization parameters. The impact of each hyperparameter and model variation is detailed in the results section for selection of the best performing model.

A program was developed to facilitate the rapid modification of the pre-trained model and the incorporation of new layers. During the training of the DL models, initial hyperparameters were configured, and training commenced. Following the training phase, if evaluation results indicated overfitting, two adjustments were made. First, efforts were made to increase the dataset size and improve its quality through data cleaning procedures. Second, hyperparameters related to regularization, such as dropout and L1 and L2 regularization methods, were modified. If overfitting was not observed, the model’s accuracy was assessed. If the accuracy was deemed unsatisfactory, adjustments were made to the hyperparameters. If this proved ineffective, modifications were made to the architecture of the added layers. This iterative process was conducted across multiple pre-trained models to identify the optimal model architecture.

The last layer of the best performing model (DenseNet 121) was eliminated for the CLF identification model. Following this, a pooling layer was inserted after the final convolution layer, which was succeeded by the addition of five dense layers. These dense layers incorporated dropout values of 0.5, 0.7, 0.5, 0.7, and 0.7, along with ReLU activation functions. The last layer comprised three outputs with a SoftMax activation function. The Adam optimizer was utilized to optimize the weights and biases of the DenseNet 121, employing a learning rate of 0.000125. The dataset was partitioned into training, validation, and testing sets in a ratio of 3:1:1.

During the training phase of each model, configurations were implemented to cease the training process if the gradient descent algorithm failed to converge after a specified number of epochs. This prevents the models from undergoing unnecessary epochs due to a lack of convergence. Additionally, each model was programmed to save its optimal version. Therefore, even if the model’s testing accuracy decreases at the cessation of training, the model has already stored the best instance with the highest accuracy.

The model was evaluated using Area Under the Curve–Receiver Operating Characteristics (AUC–ROC), precision, and recall, along with overall accuracy and loss, to ensure comprehensive assessment despite the imbalanced dataset (refer to the Supplementary Materials).

## 4. Experimental Results and Analysis

### 4.1. Quality Inspection Model

**Figure 8:**
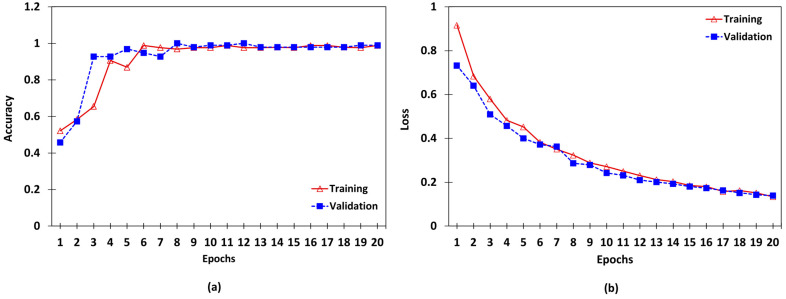
Accuracy and loss of training and validation of the quality inspection model. (**a**) Variation of accuracy with epochs. (**b**) Variation of loss with epochs.

Table 3 compares the models utilized for quality inspection, which involves classifying the input data as either low-detailed or high-detailed images. Accuracy was selected as the primary metric to assess the model performance because the training dataset was balanced. Due to its superior accuracy, the VGG-16 model was the most effective of the three models tested (VGG-16, InceptionV3, and Inception-ResNet-V2). The model reached convergence after approximately 20 epochs, resulting in a loss of 0.1608. While the other three models also demonstrated accuracy levels close to VGG-16, some base models like DenseNet-121 and ResNet-50 exhibited over fitting, converging at fewer epochs. L1 regularization was implemented on the final layer (output sigmoid layer) with a value of 0.01 to address over fitting. Although dropouts can also be employed to address over fitting, increasing other learning parameters and adding new layers with dropouts may exacerbate over fitting in this particular use case. Figure 8 illustrates the model convergence during the training of the VGG-16 model.

The model architecture was tested with other pre-trained models used in the CLS detection model. However, these models tended to overfit quickly compared to the selected three backbone models. Despite the model performing exceptionally well with the current dataset, its effectiveness is limited by the lack of diverse data for accurately identifying low-detail or noisy images. The dataset lacks different categories of noisy images that were taken while scanning the samples, in addition to several augmentations being made to it, such as flipping and adding synthetic noise. Incorporating a variety of noisy images will aid in mitigating over fitting during the training phase. Transfer learning leverages pre-trained models, which have already been exposed to a wide range of features and patterns, making them more robust and effective in handling unseen datasets.

### 4.2. Circular Leaf Spot Disease Detection Model

The comparative testing results of different baseline models employed in the development of the CLS disease stage identification model are presented in Table 4. The evaluation metrics used to assess the effectiveness of these models for CLS stage classification include OA, AUC–ROC for each class compared to others, micro-average AUC–ROC, precision, and recall.

**Figure 9:**
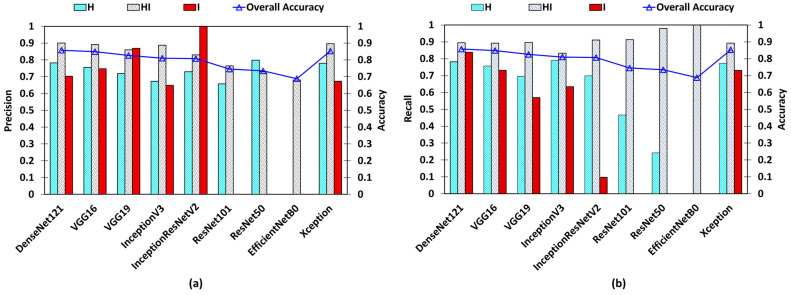
Overall accuracy (OA) with precision and recall for CLS detection model. (**a**) Precision of each class for adapted pretrained models. (**b**) Recall of each class for adapted pretrained models.

Figure 9 depicts the comparison of OA among the models. As indicated in the chart, all models achieved an accuracy of 65%, with DenseNet-121 and the Xception base models exhibiting the highest accuracies, surpassing the 85% threshold. However, due to the imbalance in the dataset, a definitive decision regarding the best model cannot be made, similar to the findings observed in the quality inspection model.

**Figure 10:**
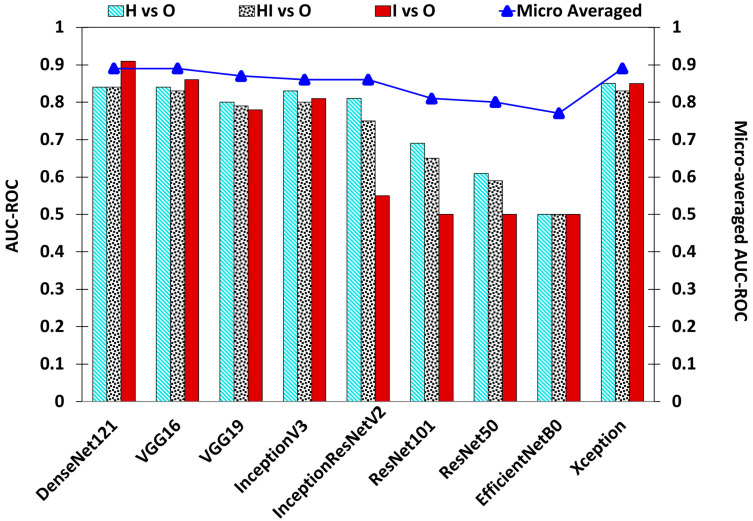
AUC–ROC calculated for predictions given by the analyzed deep learning (DL) models developed for classification of the stages of CLS disease.

An AUC–ROC is used to assess the adequate performance of a classification model, specifically in the context of binary classification tasks. Hence, the AUC–ROC was calculated for each class compared to the other two classes in the testing dataset, aiming to demonstrate the model’s performance for each class explicitly. Figure 10 displays the plot extracted from Table 4. A value of 0.5 signifies the poorest model performance, while 1 indicates the best performance in classifying each class. Notably, EfficientNetB0 exhibits the lowest performance, followed by ResNet-50 and ResNet-101. Conversely, DenseNet-121, VGG-16, VGG-19 models, InceptionV3, and Xception-based models demonstrate AUC–ROC values exceeding 0.75. The highest AUC for “H vs. Others” is observed in the Xception model at 0.85, followed by DenseNet-121 at 0.84. In addition, DenseNet-121 achieves the highest AUC–ROC values of 0.84 and 0.91 for the “HI vs. Others” and “I vs. Others” scenarios, respectively. The micro-average AUC–ROC for each model is also depicted in Figure 10. According to the chart, all of the values surpass 0.75, with the DenseNet-121 and Xception base models exhibiting the highest values.

The precision and recall metrics complement the insights provided by the AUC–ROC analysis for one class versus others. As depicted in Figure 9, ResNet-50 achieves the highest precision, close to 0.8, for class-H. However, the recall value for the ResNet-50 model is notably lower, at less than 0.25. This indicates that while the model correctly identifies a substantial proportion of samples belonging to class-H out of all of its predictions, it also overlooks a significant number of actual class-H samples that should have been identified as such. Similarly, InceptionResNetV2 exhibits the highest precision for class-I, but it faces a similar challenge as ResNet-50 with class-H, as evidenced by its very low recall value of 0.0967. On the other hand, ResNet-50, ResNet-101, and EfficientNetB0 struggle to accurately identify class-I. EfficientNetB0 demonstrates a precision of 0 for all classes and a recall value of 1 for class-HI, indicating an inability to correctly classify any class, leading to an output of HI for any input.

Although the OA and micro-averaged AUC–ROC suggest relatively similar performances across all models, it is imperative to consider additional metrics when dealing with imbalanced datasets to identify the best-performing model accurately. DenseNet-121, VGG16, and Xception-based models exhibit higher precision and recall than the others. Notably, DenseNet-121 stands out with the highest precision values of 0.7823 for class-H, 0.9005 for class-HI, and 0.7027 for class-I, supported by the highest recall values of 0.8953 for class-HI and 0.8387 for class-I, as detailed in Table 4. Consequently, DenseNet-121 has been identified as the optimal model for CLS stage identification.

**Figure 11:**
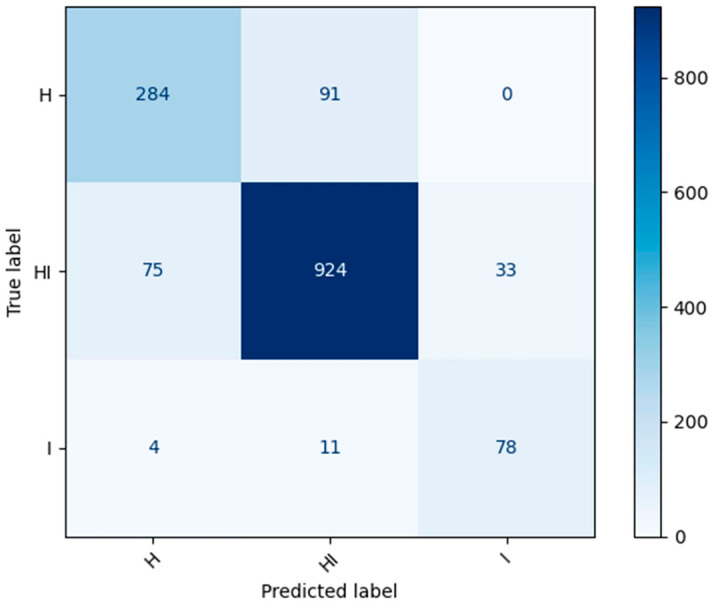
Confusion matrix of best deep learning (DL) model trained for circular leaf spot (CLS) stage identification.

**Figure 12:**
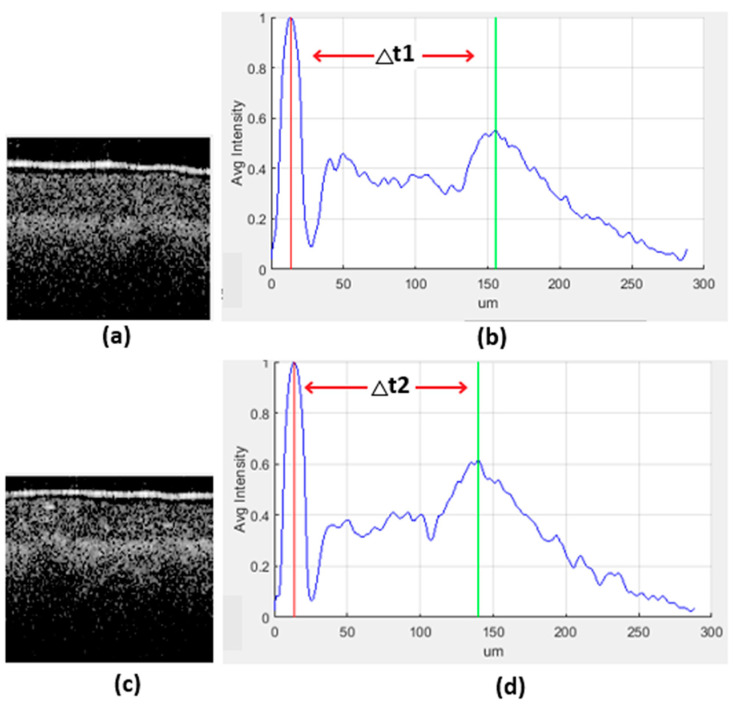
B-scan images and respective A-scan profiles of scanned leaf samples. (**a**) B-scan image of a sample labeled as H. (**b**) A-scan profile of the B-scan labeled as H because the peak difference (∆t1) is 141 µm and labeled as H by LAMP analysis. (**c**) B-scan image of a sample labeled as HI. (**d**) A-scan profile of the B-scan is labeled as HI because the peak difference (∆t2) is 126 µm and labeled as I by LAMP analysis.

The generated confusion matrix depicted in Figure 11 illustrates the model’s proficient identification of a substantial number of testing samples across the H, HI, and I classes. Notably, the model successfully avoided misclassifying any infected leaf samples (class-I) as healthy (class-H), a critical aspect given the imperative need for accurate disease diagnosis. However, approximately 10% of class-HI samples were erroneously classified as class-H. This misclassification is attributed to instances where there exists minimal discernible difference between the H and HI stages of the disease. An example of such instances is presented in Figure 12, showcasing the subtle disparities between H and HI classifications. Conversely, misclassifying H as HI can similarly occur due to the aforementioned minimal differentiations between these stages.

## 5. Discussion

The primary focus of this investigation was to develop a method for identifying three stages of CLS, namely healthy (class-H), healthy infected (class-HI), and infected (class-I), in persimmon leaves, with a subsequent emphasis on pre-identification of the disease. To achieve this objective, a detection pipeline was formulated utilizing DL techniques integrated with SD-OCT. While prior studies have examined CLS disease diagnosis [2,4], there is a scarcity of research that focuses on using OCT for this purpose [3]. The proposed DL-based pipeline demonstrates promising diagnostic performance for automated identification of CLS disease and exhibits notable proficiency in distinguishing between its different stages. It is worthy to note that the results acquired through our previously published work [3] verified the thickness threshold ranges to confirm the physical state of the leaf by measuring the thickness difference between the first two peaks of the A-scan profiles, which represent the epidermal and spongy parenchyma cell layers. Although the statistical values and quantifications such as the average, standard deviations, and minimum and maximum fluctuation range were approximated, confirming the accuracy of the study was a challenge due to the limited data analysis of a small number of amplitude scan analyses and the subjectivity of the A-scan selection. To address this major shortcoming, the proposed artificial intelligence (AI) incorporated fully automated diagnosis of CLS aims to reduce the subjectivity and time-consuming process involved in interpreting A-scan profiles generated from OCT images for CLS identification by employing deep learning techniques, which eventually confirmed a higher number accuracy that the previously conducted study.

Although DL-based methods are used to discern between infected and non-infected cases, which is highly critical in most practical agricultural contexts, circular leaf spot disease (CLS) has a long incubation period of 90–120 days, during which leaves show no symptoms until the disease is widespread. Since the apparently healthy (HI) stage is indistinguishable from the healthy stage (H) by the naked eye, identifying the disease at the HI stage is crucial for effective disease management before it becomes severe. To overcome the limitations of histological analysis, PCR, and LAMP, OCT was incorporated as a non-destructive imaging method for the early identification of CLS. Although (H) stage and (I) stage are visually distinguishable, the differentiation between (H) and (HI) is highly challenging. Also, the reliable detection of the ‘H’ class is important to avoid unnecessary exposure to pesticides, and ‘HI’ class is important for the pre-identification of the disease spread. Therefore, the proposed DL approach enables the differentiation among all three classes (‘H’, ‘HI’, and ‘I’) to ensure accurate and efficient disease management. The incorporation of both manual A-scan profile analysis and LAMP analysis into the DL approach resulted in higher accuracy compared to using manual A-scan analysis alone. Moreover, this study also marks the first use of a combination of LAMP with A-scan for the early detection of CLS disease.

The integration of OCT and DL for automated classification within agricultural contexts remains uncommon. Prior work by Josh et al. [39] focused on rice seed classification employing a modified Inception-ResNet-V2 model, while Manhando et al. investigated early detection of mold-contaminated peanuts using a modified VGG-16 model, yielding promising outcomes. In contrast, this study is the first attempt to use OCT with DL to automatically identify diseases that spread across plant leaves. However, extensive research has been undertaken regarding the automated diagnosis of medical conditions using OCT and DL. The utilization of the DenseNet-121 and VGG-16 models used in this study resulted in positive outcomes within the medical domain [52,53,54,55,56].

In this study, two CNN models were constructed, leveraging pre-trained DL models renowned for their efficacy in handling small datasets. Although the primary focus of the study pertains to the identification of CLS stages, a supplementary quality inspection model was introduced following pre-processing to mitigate the impact of low-detail images on CLS disease identification. This addition notably enhanced the performance of the CLS detection model. The modified DenseNet-121 model developed for CLS detection demonstrated promising performance, particularly in precision across all classes and recall values for specific classes, resulting in an impressive overall AUC–ROC of 0.89. The evaluation of quality inspection models revealed that the modified VGG-16 model achieved the highest accuracy of 98.99% among various pre-trained models, specifically tailored for discerning low and high detailed images.

Overall, the proposed models demonstrate a robust capacity to effectively discern classes HI and I with elevated recall (sensitivity) according to Figure 11, thereby reducing the risk of erroneously categorizing diseased leaves as healthy. This aspect holds significant importance for farmers, as it enables prompt initiation of treatments for afflicted crops.

The predictive accuracy of the employed DL models in the study could be enhanced through various means. A primary limitation lies in the scarcity of the dataset, as DL models typically require extensive datasets to effectively train their parameters [41]. Furthermore, not all of the images in the dataset could be taken into consideration due to their poor level of detail. Additionally, inconsistencies in the labeling procedure were observed when comparing results obtained from LAMP tests and A-scan profile analysis. There were several instances that were not taken into consideration since the LAMP result suggested class-H, but the A-scan result indicated class-I. These inconsistencies can be attributed to differences in the physical thickness of leaves across different scanning locations. The model is customized for the OCT scanning data used in this study but faces challenges in generalizing to accommodate diverse images from different devices and conditions, similar to issues in other imaging modalities [57]. As a result, efforts are needed to enhance the model’s ability to generalize across various datasets with distinct properties and acquisition conditions.

The suggested framework for the early detection of CLS holds promise for integration into an OCT scanning system, particularly one utilized in prior investigations by Wijesinghe et al. [21]. By incorporating this framework into a handheld scanner, the capability for real-time, in situ disease identification in field settings would be enhanced, facilitating the development of a farmer-centric product for automated disease detection with high accuracy, eliminating the necessity for expert knowledge in plant diseases.

## 6. Conclusions

In conclusion, this study developed a detection pipeline for the pre-identification of CLS in persimmon leaves by integrating DL techniques with SD-OCT. While previous studies have explored manual CLS disease diagnosis, this investigation represents a pioneering effort to automate CLS identification, addressing the issues of time-consuming analysis and subjectivity found in earlier methods. The image labeling process, incorporating both A-scan analysis and LAMP results, significantly enhanced the accuracy for efficient pre-identification of CLS compared to manual analysis relying solely on A-scan profiles.

The application of CNNs, particularly the adapted DenseNet-121 and VGG-16 models, displayed promising diagnostic capabilities. The adapted DenseNet-121 model excels in differentiating between three stages of CLS disease in persimmon leaves, including healthy (class-H), healthy-looking (class-HI), and infected (class-I), specifically focusing on the pre-identification of CLS through the “healthy-looking” class. This adapted DenseNet-121 model achieved commendable precision values of 0.782 for class-H, 0.9 for class-HI, and 0.702 for class-I, supported by the highest recall values of 0.8953 for class-HI and 0.838 for class-I. The performance of CLS detection was further boosted by the introduction of a supplemental quality inspection model adapted using VGG-16, which achieved an accuracy of 98.99% in discriminating between low-detailed images and high-detailed images.

This research highlights the capacity of DL techniques combined with OCT to improve disease identification procedures, particularly within agricultural contexts. By offering effective and objective pre-identification of CLS, this study enables early intervention and management approaches in persimmon cultivation.

## Figures and Tables

**Table 1:** Difference between peaks of A-scan profiles for labeling the CLS stages.

Class Label	Ranges in µm (∆*t*)
H-healthy	140+
HI-apparently healthy	90–130
I-infected	0–89

**Table 2:** Labeling procedure of H, HI, and I based on LAMP and A-scan analysis.

A-Scan Label	LAMP Label	Class Label
H	H	H
HI	I	HI
I	H	H
H	I	HI
HI	H	H
I	I	I

**Table 3:** Testing results of quality inspection models trained over multiple pre-trained models.

Base Model	Epochs	Loss	Accuracy
VGG16	20	0.0608	0.9899
InceptionResNetV2	20	0.1088	0.9828
InceptionV3	50	0.1022	0.9885

**Table 4:** Testing results of CLS classification models trained over multiple pre-trained models.

Base Model	Epochs	Learning Rate	OL	OA	AUC–ROC				Precision			Recall		
					H vs. O	HI vs. O	I vs. O	Micro Averaged	H	HI	I	H	HI	I
DenseNet121	110	0.000125	0.3249	0.8573	0.84	0.84	0.91	0.89	0.7823	0.9005	0.7027	0.7573	0.8953	0.8387
VGG16	114	0.0001	0.369	0.8487	0.84	0.83	0.86	0.89	0.7553	0.8915	0.7472	0.7573	0.8924	0.7311
VGG19	111	0.0001	0.3861	0.826	0.8	0.79	0.78	0.87	0.719	0.8596	0.8688	0.696	0.8963	0.5698
InceptionV3	82	0.0001	0.4391	0.81	0.83	0.8	0.81	0.86	0.6727	0.8875	0.6483	0.7893	0.833	0.6344
InceptionResNetV2	109	0.0001	0.4317	0.8073	0.81	0.75	0.55	0.86	0.7298	0.8303	1	0.6986	0.9108	0.0967
ResNet101	68	0.00001	0.5815	0.7453	0.69	0.65	0.5	0.81	0.6578	0.7641	0	0.4666	0.9137	0
ResNet50	86	0.00001	0.5816	0.7347	0.61	0.59	0.5	0.8	0.7982	0.7294	0	0.2426	0.9796	0
EfficientNetB0	122	0.0001	0.7763	0.688	0.5	0.5	0.5	0.77	0	0.688	0	0	1	0
Xception	86	0.000125	0.3514	0.8527	0.85	0.83	0.85	0.89	0.7795	0.8967	0.6732	0.7733	0.8924	0.7311

## Data Availability

The raw data supporting the conclusions of this article will be made available by the authors on request.

## Supplementary Materials

The following supporting information can be downloaded at: https://www.mdpi.com/article/10.3390/s24165398/s1.

## Abbreviations

AMD	Age-related macular degeneration
ANN	Artificial neural networks
AUC	Area under the curve
BN	Batch normalization
CLS	Circular leaf spot
CNN	Convolutional neural network
CUDA	Compute unified device architecture
DL	Deep learning
DME	Diabetic macular edema
DNN	Deep neural network
FPR	False positive rate
GPU	Graphical processing unit
GUI	Graphical user interface
LAMP	Loop-mediated isothermal amplification
ML	Machine learning
MRI	Magnetic resonance imaging
OA	Overall accuracy
OCT	Optical coherence tomography
PCR	Polymerase chain reaction
ReLU	Rectified linear unit
RNN	Recurrent neural network
ROC	Receiver operating characteristics
SD-OCT	Spectral domain OCT
SGD	Stochastic gradient descent
TPR	True positive rate
