# Supplementary material for: Dense Convolutional Neural Network-Based Deep Learning Pipeline for Pre-Identification of Circular Leaf Spot Disease of Diospyros kaki Leaves Using Optical Coherence Tomography
Source: Sensors (Basel). 2024 Aug 21;24(16):5398. doi: 10.3390/s24165398 (PMC11359294; doi:10.3390/s24165398)
Supplement: Supplementary file 1 [file sensors-24-05398-s001.zip › sensors-3084740-supplementary.pdf]

sensors

Supplementary Materials

Dense Convolutional Neural Network-Based Deep Learning Pipeline for Pre-Identification of Circular Leaf Spot Disease of *Diospyros kaki* Leaves Using Optical Coherence Tomography

Deshan Kalupahana [1], Nipun Shantha Kahatapitiya [1], Bhagya Nathali Silva [2,3], Jeehyun Kim [4], Mansik Jeon [4], Udaya Wijenayake [1,*], and Ruchire Eranga Wijesinghe [3,5,*]

[1] Department of Computer Engineering, Faculty of Engineering, University of Sri Jayewardenepura, Nugegoda 10250, Sri Lanka; deshankalupahana@sjp.ac.lk (D.K.); egt18538@sjp.ac.lk (N.S.K.)
[2] Department of Information Technology, Faculty of Computing, Sri Lanka Institute of Information Technology, Malabe 10115, Sri Lanka; nathali.s@sliit.lk
[3] Center for Excellence in Informatics, Electronics & Transmission (CIET), Sri Lanka Institute of Information Technology, Malabe 10115, Sri Lanka
[4] School of Electronic and Electrical Engineering, College of IT Engineering, Kyungpook National University, 80, Daehak-ro, Buk-gu, Daegu 41566, Republic of Korea; jeehk@knu.ac.kr (J.K.); msjeon@knu.ac.kr (M.J.)
[5] Department of Electrical and Electronic Engineering, Faculty of Engineering, Sri Lanka Institute of Information Technology, Malabe 10115, Sri Lanka
* Correspondence: udayaw@sjp.ac.lk (U.W.); eranga.w@sliit.lk (R.E.W.)

1. Hyperparameter Tuning for Selecting the Best Performing Circular Leaf Spot Detection Model

Transfer learning in image classification involves utilizing pre-trained parameters to detect image features, which are then utilized by an FCN for classification. For instance, DenseNet121 comprises approximately 8.1 million parameters. In the tested environment, training one epoch from random initialization took 26 seconds, whereas with pre-trained weights, it only took 6 seconds. Figure S1 illustrates the training loss and accuracy of models initialized randomly versus with pre-trained weights. The chart indicates that the pre-trained model shows a gradual increase in accuracy and a corresponding gradual decrease in training loss. In contrast, the randomly initialized model exhibits a rapid initial increase in accuracy and loss reduction, indicative of overfitting. Validation curves further highlight significant fluctuations in the randomly initialized model, exceeding a variance threshold of 1, indicating inconsistent results on unseen data. These results arise because the model rapidly memorizes the training data instead of learning generalizable features. This overfitting is due to the absence of prior knowledge provided by pre-trained weights, leading the model to concentrate on the specifics of the training data, which results in poor performance on unseen data. Therefore, the study proceeded with the pre-trained model due to its stability and superior performance on validation metrics.

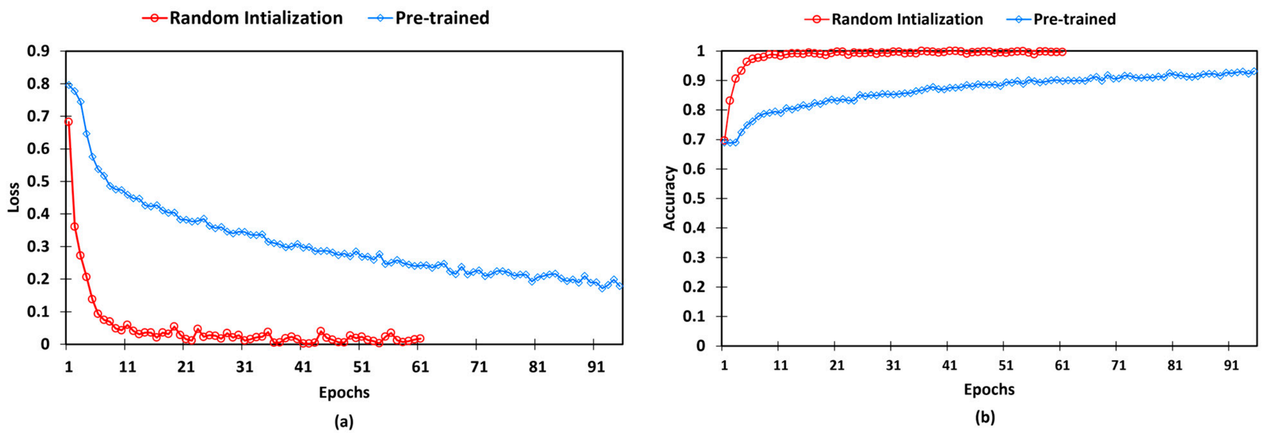

Figure S1. Training loss and accuracy of DenseNet121 model with random initialization and pre-trained weights. Curves for the randomly initialized model exhibited a greater tendency to overfit compared to the pre-trained model. Therefore, pre-trained models were utilized for the development of the CLS detection model. (**a**) Variation of loss with epochs. (**b**) Variation of accuracy with epochs.

The number of epochs required for models to converge and the optimal learning rate for convergence were recorded. The models were configured to halt training if the validation loss remained unchanged for 50 epochs. The optimal learning rate was determined by observing the point at which the training loss attained its smallest value for a given learning rate. The TensorFlow Keras learning rate scheduler was employed to determine the optimal learning rate for training each DL model. The resulting learning rate for the DenseNet-121 model was determined to be 0.000125, as depicted in Figure S2.

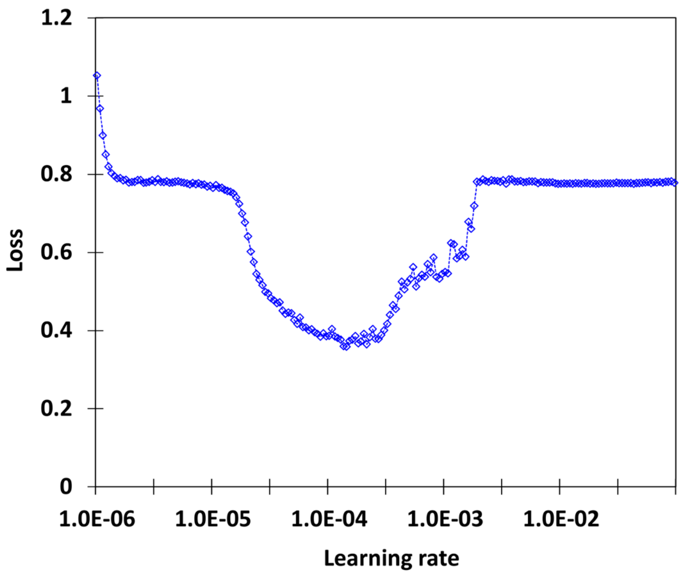

Figure S2. The relationship between training loss and learning rate was utilized to determine the optimal learning rate for training the DenseNet-121 model.

Moreover, the impact of the learning rate on validation loss and accuracy during the training of the DenseNet-121-based model is illustrated in Figure S3 and Figure S4. This demonstrates that a learning rate of 0.000125 facilitated optimal convergence of loss while achieving higher accuracy.

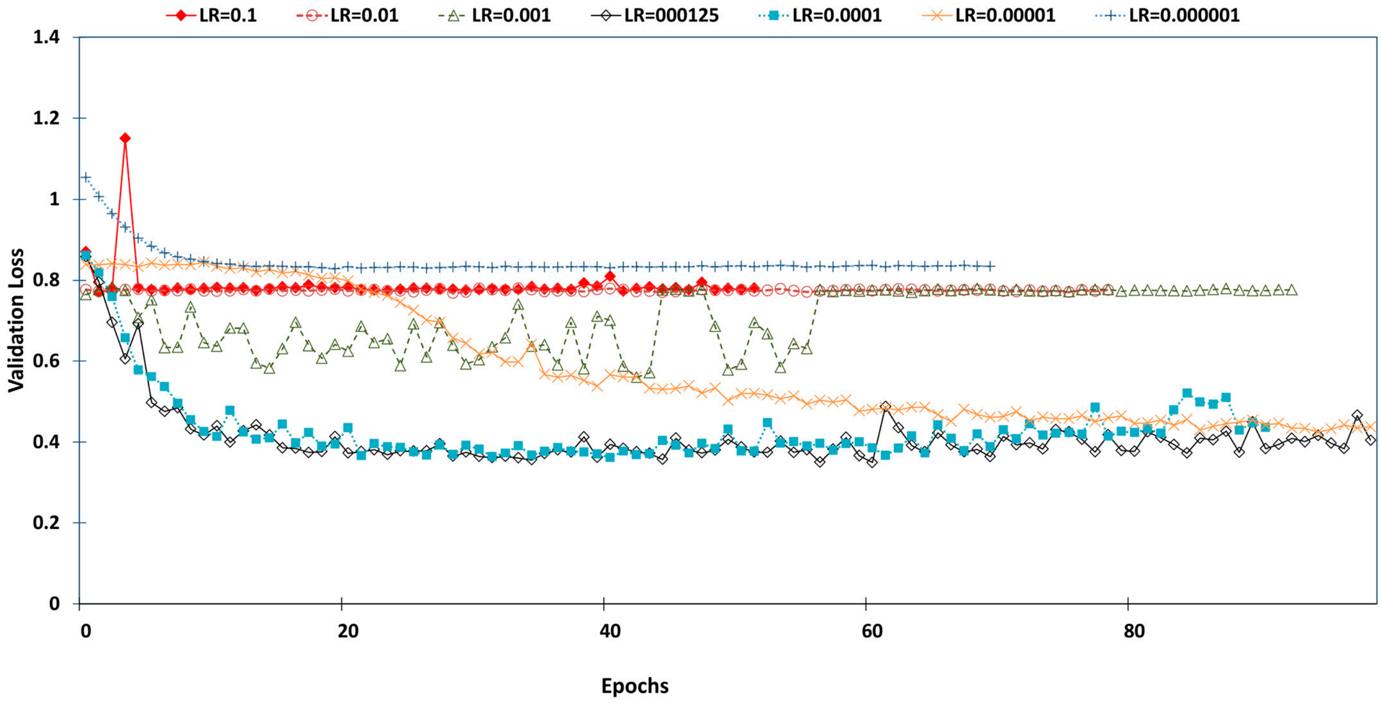

Figure S3. The variation in validation loss across different learning rates for the circular leaf spot (CLS) detection model.

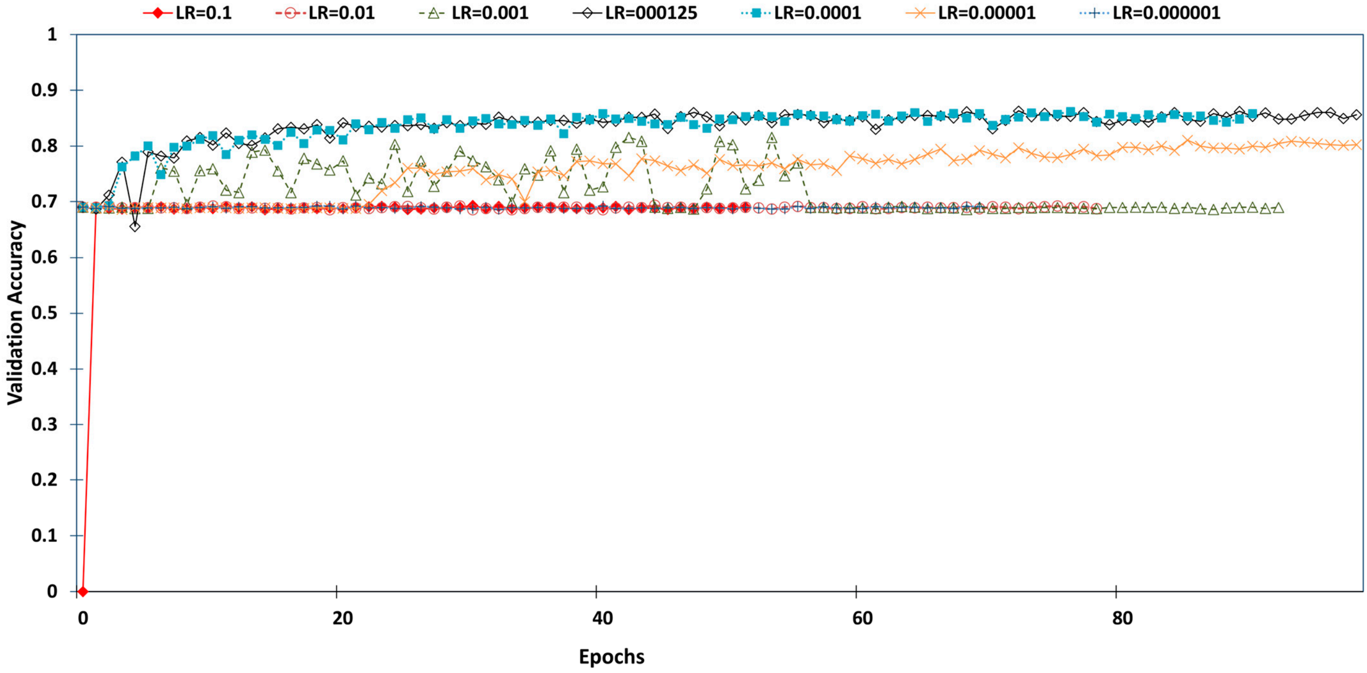

Figure S4. The variation in validation accuracy across different learning rates for the circular leaf spot (CLS) detection model.

In transfer learning, the fine-tuning of models relies heavily on adjustments made to the FCN parameters. Increasing the number of layers in the FCN leads to a greater number of learning parameters, which increases the training period and the risk of overfitting. Figure S5 illustrates the variation in training and testing accuracy and loss with different numbers of hidden layers in the FCN. The testing accuracy initially improves with an increasing number of layers but begins to decline after reaching seven layers. Regarding the loss curves, the validation loss converges consistently up to the sixth instance but shows divergence in the seventh. The FCN with six hidden layers was selected as the optimal model due to its higher accuracy in both testing and validation datasets, lower loss in these datasets, and enhanced capacity to effectively learn specific data patterns, attributed to its higher number of learning parameters.

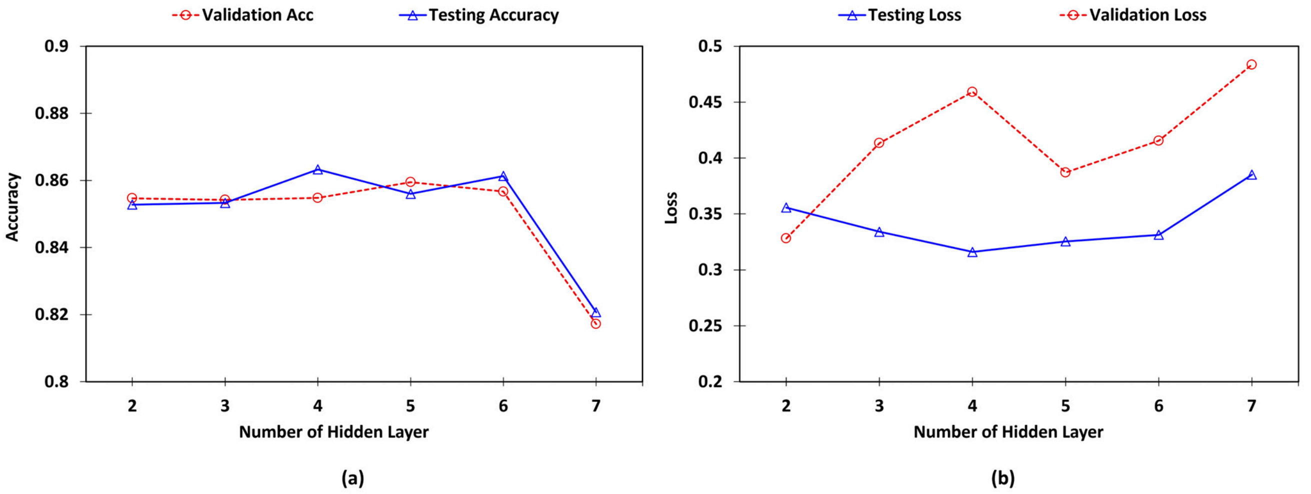

Figure S5. Variation of validation and testing accuracy and loss according to the number of hidden layers of the Dense121-based model. The model with six hidden layers demonstrated high accuracy in both validation and testing datasets, along with comparatively low loss in both phases. Therefore, this configuration was chosen as the FCN configuration for the developed model. . (a) Variation of accuracy with number of hidden layers. (b) Variation of loss with number of hidden layers.

The impact of batch size on the performance of the CLS detection model is illustrated in Figure S6. Testing accuracies remain consistent, approximately 0.85 across various batch sizes, indicating minimal variation. Testing loss plateaued around 0.35 and increased beyond a batch size of 256. The tested DenseNet121 based model achieved optimal results with a batch size of 16, along with other hyperparameter values. The consistency in testing accuracies suggests that the model has reached a level of generalization, performing reliably well regardless of batch size. This indicates the model's resilience to batch size variations, maintaining stable accuracy across different configurations.

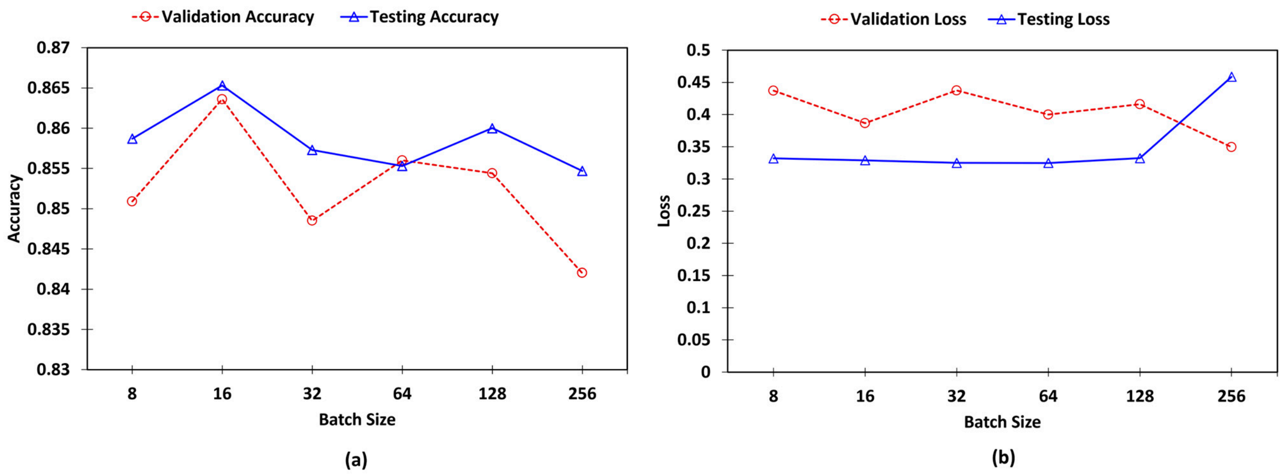

Figure S6. Variation of validation and testing accuracy and loss according to the batch size of the Dense121 based model. The curves did not show significant variation between different batch sizes. However, a batch size of 16 yielded the best results, demonstrating higher accuracy and lower loss in the model. (a) Variation of accuracy with batch size. (b) Variation of loss with batch size.

Dropouts are integrated into the model as a means of regularization to prevent overfitting. Figure S7 illustrates the impact of dropouts on the performance of the best-performing DenseNet-121 model. Without dropouts in the hidden layers, models tend to overfit, evidenced by significant fluctuations in the validation curves. The inclusion of dropouts reduces these fluctuations by randomly deactivating a portion of neurons during training. This technique promotes better generalization of unseen data by discouraging the network from overly relying on specific neurons or features present in the training data. Various dropout values were tested in the FCN, with specific values of 0.5, 0.7, 0.5, 0.7, and 0.7 chosen for the six-layer FCN configuration in the optimized DenseNet-121 model. L1 and L2 regularizations were not employed in this model, as the desired balance between accuracy and loss curves was achieved through careful adjustment of dropout rates.

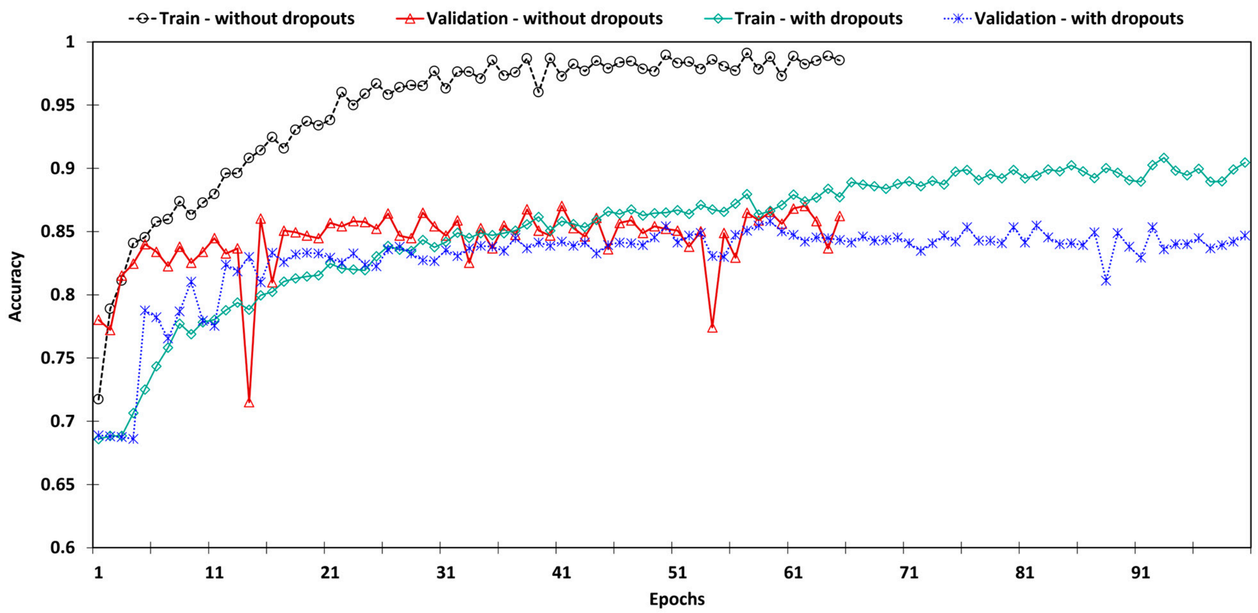

Figure S7. Variation of training and validation accuracy with and without dropouts in FCN. The curves without dropouts tend to overfit quickly, exhibiting large fluctuations in the validation

curve. In contrast, models with dropouts show smoother stabilization in both training and validation curves. Therefore, dropout was applied to the developed model to enhance stability and performance

Batch size, number of layers, dropouts, and learning rates were fine-tuned to optimize the DL model after selecting the best-performing configuration. As indicated earlier, changes in these hyperparameters did not lead to significant differences in performance. Throughout the study, various datasets were created using different pre-processing techniques to enhance dataset quality. The pre-processing steps applied to the dataset used for the best-performing model are detailed in the methodology section. Table S1 shows the improvement of DL models with enhanced datasets over time for the best-performing models.

Table S1. Accuracy and loss of testing, validation, and training for major dataset versions employed in the study.

Dataset	Test		Final Validation		Final Training	
Version	Loss	Accuracy	Loss	Accuracy	Loss	Accuracy
2022_feb	0.5697	0.7899	0.5616	0.7853	0.5342	0.7627
2022_july	0.5817	0.7748	0.5804	0.7857	0.5265	0.7551
2022_sept	0.6259	0.8673	0.8102	0.8125	0.0471	0.9831
2022_oct	0.4607	0.8158	0.4573	0.8261	0.2186	0.8261
2023_may	0.3249	0.8613	0.4375	0.8485	0.1783	0.9279

2. Hyperparameter Tuning for Selecting the Best Performing Circular Leaf Spot Detection Model

Classification performance is assessed using various metrics, with both models employing overall accuracy (OA). Due to the dataset's imbalance in CLS stage classification, supplementary evaluation metrics, including Area Under the Curve Receiver Operating Characteristics (AUC-ROC) One vs All and micro-averaged AUC-ROC, precision, and recall were computed. These metrics are derived from the combinations of True Positive (TP), True Negative (TN), False Positive (FP), and False Negative (FN) values. Equations for OA and precision are given by Equation (S1) and Equation (S2), respectively.

$$OA = \frac{TP + TN}{TP + TN + FP + FN} \tag{S1}$$

$$Precision = \frac{TP}{TP + FP} \tag{S2}$$

An AUC-ROC provides a visual depiction of the performance of a binary classification model across different classification thresholds. It presents the True Positive Rate (TPR), commonly referred to as sensitivity or recall, on the vertical axis and the False Positive Rate (FPR) on the horizontal axis. TPR and FPR are presented in Equation (S3) and Equation (S4).

$$Recall = TPR = \frac{TP}{TP + FN} \tag{S3}$$

$$FPR = \frac{FP}{FP + TN} \tag{S4}$$

Micro-averaging involves consolidating predictions for each specific class and then calculating an AUC-ROC curve using the amalgamated predictions. Equations for calculation of TPR and FPR for micro-averaged AUC-ROC is given by Equation (S5) and Equation (S6) respectively.

$$TPR = \frac{\sum_c TP_c}{\sum_c (TP_c + FN_c)} \quad \text{(S5)}$$

$$FPR = \frac{\sum_c FP_c}{\sum_c (FP_c + TN_c)} \quad \text{(S6)}$$
